# Long-term reduction in hyperglycemia in advanced type 1 diabetes: the value of induced aerobic glycolysis with BCG vaccinations

**DOI:** 10.1038/s41541-018-0062-8

**Published:** 2018-06-21

**Authors:** Willem M. Kühtreiber, Lisa Tran, Taesoo Kim, Michael Dybala, Brian Nguyen, Sara Plager, Daniel Huang, Sophie Janes, Audrey Defusco, Danielle Baum, Hui Zheng, Denise L. Faustman

**Affiliations:** 1 Immunobiology Laboratories, Massachusetts General Hospital and Harvard Medical School, Bldg 149, 13th Street, Boston, MA 02116 USA; 2 Department of Biostatistics, Massachusetts General Hospital, Boston, MA 02115 USA

## Abstract

*Mycobacterium* are among the oldest co-evolutionary partners of humans. The attenuated *Mycobacterium bovis* Bacillus Calmette Guérin (BCG) strain has been administered globally for 100 years as a vaccine against tuberculosis. BCG also shows promise as treatment for numerous inflammatory and autoimmune diseases. Here, we report on a randomized 8-year long prospective examination of type 1 diabetic subjects with long-term disease who received two doses of the BCG vaccine. After year 3, BCG lowered hemoglobin A1c to near normal levels for the next 5 years. The BCG impact on blood sugars appeared to be driven by a novel systemic and blood sugar lowering mechanism in diabetes. We observe a systemic shift in glucose metabolism from oxidative phosphorylation to aerobic glycolysis, a state of high glucose utilization. Confirmation is gained by metabolomics, mRNAseq, and functional assays of cellular glucose uptake after BCG vaccinations. To prove BCG could induce a systemic change to promote accelerated glucose utilization and impact blood sugars, murine data demonstrated reduced blood sugars and aerobic induction in non-autoimmune mice made chemically diabetic. BCG via epigenetics also resets six central T-regulatory genes for genetic re-programming of tolerance. These findings set the stage for further testing of a known safe vaccine therapy for improved blood sugar control through changes in metabolism and durability with epigenetic changes even in advanced Type 1 diabetes.

## Diabetes: Repurposing a classic tuberculosis vaccine

In patients with long-term type 1 diabetes, the tuberculosis vaccine BCG lowers blood sugar levels to near-normal after three years. Denise Faustman and her team from Massachusetts General Hospital and Harvard Medical School investigated a cohort of type 1 diabetics that received two doses of BCG before being monitored over eight years. After three years, vaccine-treated patients lowered their HbA1c levels—a diabetes biomarker reflecting average blood sugar over 8–12 weeks—by over 10%. This reduction increased to 18% in the fourth year, after which HbA1c levels remained low up to the final year of monitoring. The researchers report that the BCG vaccine appeared to reset diabetes-implicated parts of the immune system and, through a novel mechanism, shift glucose metabolism to lower blood sugar to healthy levels. Future studies will further classify BCG’s benefits in diabetes.

## Introduction

The bacillus Calmette-Guérin (BCG) vaccine is one of the oldest vaccines in the world, developed for tuberculosis (TB) protection and for early stage bladder cancer therapy. BCG is an attenuated version of the virulent *Mycobacterium bovis*. The avirulent *Mycobacterium bovis* is a close relative of pathologic *Mycobacterium leprae* and *Mycobacterium tuberculosis*. The co-evolution of humans and *Mycobacterium* is not recent but extends back some 90,000 years.^1^ This is the longest time span for any identified pathogen co-evolution. It is essential to understand the adaptive changes of the human genome to such prolonged coevolution and the impact of re-introduction of this attenuated form of this microorganism.^2^

The past 10 years has seen a surge of clinical trials that re-introduce BCG for a diversity of autoimmune, allergic, and induced adaptive immune responses to childhood infections.^3–11^ In multiple sclerosis, BCG halts new onset disease, yet the clinical effect is most dramatic nearly 5 years later.^12^ In type 1 diabetes (T1D), three BCG vaccines administered in childhood lowered the incidence of T1D by age 12.^10^ In a Phase I trial with multi-dosing BCG in long-term T1Ds promising biomarker responses such as increased beneficial T-regulatory (Treg) cells and transient restoration of small amounts of pancreatic insulin were observed, but at the end of the 20-week trial, the established clinical marker for disease reversal, lowered hemoglobin A1c (HbA1c), was not observed.^4^ BCG vaccination confers a survival advantage in low birthweight infants against mortality from a diversity of infections unrelated to tuberculosis, and BCG vaccinations in healthy populations confer long term survival advantages.^13–16^

Many autoimmune NOD (non-obese diabetic) murine studies have shown a beneficial effect of BCG or CFA (Complete Freund’s Adjuvant) in preventing the onset of autoimmune diabetes and even reversing full blown established disease in mice.^17,18^ The NOD mouse is a well-studied spontaneous model of autoimmune diabetes and mimics some but not all the features of human disease.^19^ Prior data has also taught us important lessons on BCG dosing and the timing of BCG administrations in BCG efficacy.^17,18,20–24^ The commonly used NOD mouse model of spontaneous autoimmune diabetes illustrates the value of BCG for both disease progression, early disease treatment, and remarkably even disease reversal in advanced murine diabetes, an uncommon result for immune interventions.^17,18,20–22^ If NOD mice are administered BCG after the NOD mice exhibit signs of diabetes, i.e., pre-diabetes, new onset diabetes, or full-blown diabetes, the BCG permanently cures diabetes. In contrast, administration of BCG at birth in diabetes-prone humans or NOD mice as a single injection has no or a detrimental effect, so the disease must be present and the underlying autoimmune state allows BCG to be effective in mice and humans when the autoimmunity is ongoing in genetically prone models^17,18,20–24^

The lessons learned from mouse studies include the increased efficacy of multi-dosing BCG when the disease is apparent and the variable efficacy of different BCG strains.^25^ These lessons have been mirrored by human studies as well. A single dose of the *Moreau* BCG substrain appears to decrease new onset human diabetes progression, but three subsequent human studies using less potent BCG strains, such as *TICE*, demonstrated no clinical effect in humans, at least with the limited follow-up times utilized in these studies.^26–28^ The *TICE* strain of BCG for instance is known to have poor immune regulatory properties in both NOD mice and humans and in culture experiments has weakened efficacy for TNF and NF*k*B induction.^27^ This published data illustrates the importance of BCG dose, BCG strain, and timing of BCG administration as critical for murine autoimmune disease reversal and maybe even early signs of efficacy or failed efficacy in human type 1 diabetes.

The rational of this study was to investigate in established T1D subjects (average disease duration of 19 years), the possible long term immune, metabolic and clinical benefits of two doses of BCG vaccine as the Connaught strain (given two times, 4 weeks apart). This 8-year-long Phase 1 trial shows the long-term lowering of blood sugars after year 3 measured by HbA1c. The lowering of blood sugars to a range near normal was maintained for the next 5 years of monitoring. We mechanistically dissect these durable clinical results. Repeat BCG appears to reset the immune system by de-methylating in CD4 T cells all six key T-regulatory genes by 8 weeks, resulting in enhanced mRNA expression, according to epigenetic analysis. T regulatory cells are critical for immune balance and are believed to facilitate a decrease in inflammation and to prevent or slow the autoimmune process. BCG appears to switch the immune system of T1Ds from high oxidative phosphorylation to augmented early aerobic glycolysis, a systemic metabolic shift that occurs gradually and allows cells to consume in a regulated fashion large amounts of glucose to safely lower hyperglycemia to near normal levels. Taken together, the findings suggest the benefit of targeted reintroduction with attenuated *Mycobacterium bovis* as a BCG vaccine for blood sugar control through epigenetics and altered metabolism.

## Results

This is a study of 282 human research participants for both in vivo BCG vaccine clinical trial studies (*n* = 52) and in vitro mechanistic studies (*n* = 230). Of these total research subjects 211 had type 1 diabetes and 71 were non-diabetic control subjects. The details of all subjects and corresponding clinical subject data are depicted in Supplementary Table S1.

### Long-term and stable blood sugar reduction with BCG vaccinations

BCG-vaccinated T1Ds were compared to a placebo group as well as a reference group of T1Ds receiving standard of care at the Massachusetts General Hospital in part reported after 20 weeks in the Phase I double blinded clinical trial^4^ (Supplementary Table S1b). We extended this trial with 8 year long monitoring (Supplementary Table 1b—8 year long subjects) and additional in vivo BCG treated subjects (Supplementary Table S1a—up to 5 year long subjects).

**Fig. 1:**
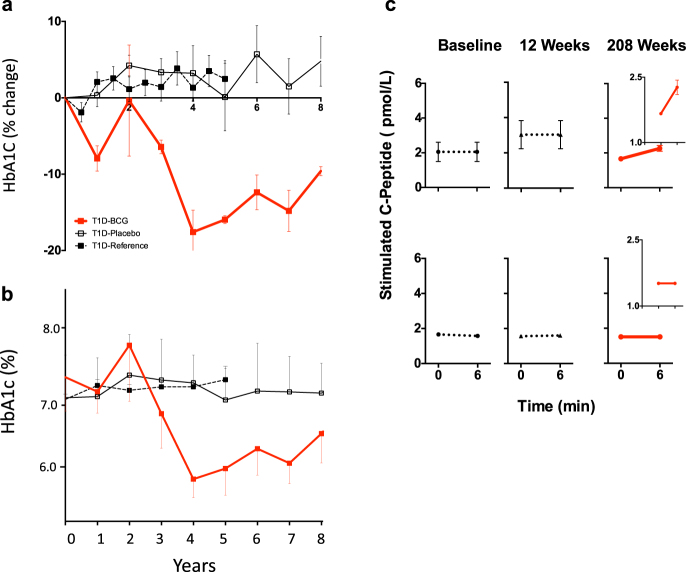
Long-term improvement of glycemic control in T1Ds after BCG treatment. **a**, **b** Glucose control was tracked through measurements of HbA1c. HbA1c levels in the control T1D groups (saline-treated placebo group *n* = 3 or the untreated reference group *n* = 40) remained unchanged over the 8-year observation period (placebo) or 5-year observation period (untreated reference group) as measured by a % change (**a**) or as raw HbA1c values (**b**) (*p* = 0.73). The percentage change was calculated from pre-trial values to post-trial values measured every 6 months or yearly. In contrast, a decrease in HbA1c for the 8 year long followed BCG-treated patients uniformly occurred after year 03 and thereafter showed sustained lowering, with an 18% decrease from baseline at year 04. After the drop in HbA1c values in the BCG-treated group, HbA1c values remained lower for the next 5 years of monitoring and was statistically different from placebo (*p* = 0.0002 at year 8) and from reference subjects (*p* = 0.02 at year 5). In short, relative (percent) change rate of HbA1c was compared using the linear mixed effects model with subject-level random effects. The change rates in the control, placebo and BCG groups were compared based on the statistical significance of the interaction term between time and group indicator in the linear mixed effects model. The subject traits and sample sizes are given in Supplementary Figure S1a, S1b. **c** An in vivo glucagon stimulation test was performed to induce pancreatic insulin secretion as measured with C-peptide assays at trial enrollment (baseline), at 12 weeks and at 208 weeks after two BCG vaccinations in 6 T1D subjects (Supplementary Table S1b—8 year long treated subjects). The BCG-treated patients showed a clinically negligible, but statistically significant, return of stimulated serum C-peptide levels upon glucagon administration only at 208 weeks (upper panel), whereas the C-peptide response to glucagon in the reference-T1D and placebo-T1D groups remained unchanged (lower panel). For the glucagon stimulation test statistics, we used the Wilcoxon Signed Rank test. On all 8 year followed subjects with the data presented at year 04 i.e., 208 weeks. Figure inserts at 208 weeks after treatment highlight the minor changes and the standard error bars

The 8 year long followed and BCG-treated T1Ds showed a reduction in HbA1c levels of greater than 10% after year 03, 18% at year 04, and the HbA1c remained low for the next 5 years (*p* = 0.0002 at year 8) (Fig. 1a, b). Subject numbers and traits are given in Supplementary Table S1a, S2. In contrast, the placebo group (*n* = 3) and the reference T1D groups (*n* = 40) had consistently higher HbA1c over the entire monitoring period of 8 years and 5 years, respectively (Fig. 1a, b, Supplementary Table S2). The efficacy of BCG was also apparent with raw HbA1c values, which show the BCG-treated TIDs by year 8 declined to a HbA1c value of 6.65% (Supplementary Table S2, Fig. 1b). BCG treatment subjects with the improved and tight blood sugar control demonstrated blood sugar stability and lowered blood sugar persistently for 5 continuous years after the initial drop in values. Semi-annual surveys confirmed that during year 03 to year 08 after BCG vaccinations there were no reports of severe hypoglycemia by any patient, even with lowered HbA1Cs near the normal range, and no change in their care as it related to new insulin pumps or continuous glucose monitoring devices. The placebo group of subjects continued to show hypoglycemia events during the same time periods of monitoring.

We presumed at this point that the return of near normoglycemia in the BCG treated human T1D (8 year long followed subjects) was by the same mechanism as was observed in the NOD mouse treated with BCG, i.e., restored insulin from pancreas regeneration. In the genetically prone non-obese diabetic (NOD) mouse model of type 1 diabetes, the mechanism of stable blood sugar restoration after BCG is driven in large part by the regeneration of insulin-secreting islets in the pancreas.^17,18^ As previously published, the elevations in tumor necrosis factor (TNF) from the BCG vaccine stimulate cytotoxic T cell death and beneficial Treg expansion.^4,29,30^ We sought proof of a similar mechanism in humans of pancreatic islet regeneration as the cause for restored blood sugar control by measuring endogenous insulin secretion through the co-secreted C-peptide levels. C-peptide is co-secreted with insulin from the pancreas and can be used to selectively detect the secretion of endogenous insulin. Insulin levels cannot be used to look for pancreas regeneration since all subjects take exogenous insulin.

Stimulated C-peptide was measured with a glucagon challenge in the BCG and placebo type 1 diabetic subject groups at three time points (pre-BCG, post-BCG 12 weeks, and 208 weeks) to look for pancreas recovery or regeneration (Fig. 1c, Supplementary Table S1b). C-peptide is co-secreted with insulin from the pancreas and can be used to selectively detect the secretion of endogenous insulin. Insulin levels cannot be used to look for pancreas regeneration since all subjects take exogenous insulin.

In this study we observe the long term and stable lowering of blood sugars in humans after BCG vaccinations. In the human, this stable blood sugar control was not driven primarily in these human subjects by pancreas recovery or regeneration. The human pancreas after BCG even at four years after repeat vaccinations did not secrete significant insulin as clinically measured by C-peptide. The mechanism for lowered HbA1c values was not equivalent to the NOD diabetic mouse pancreas regeneration after BCG treatment, despite equally restored and long term improved blood sugar control. The BCG-treated type 1 diabetic subjects at year 4 after glucagon challenge had a negligible to no return of clinically significant C-peptide. The C-peptide values after glucagon were in the range of 2–3 pmol/L of C-peptide (Fig. 1c), but with no known clinical significance. Therefore we concluded that BCG vaccinations did not induce a clinically meaningful return of C-peptide levels in the pancreas by regeneration, as observed in the NOD mouse model of diabetes^17,18^ Thus pancreas rescue or regeneration could not fully account for the persistent and long term HbA1c lowering in humans receiving BCG.

### After BCG vaccinations, regulatory T cell signature genes are de-methylated in vivo resulting in enhanced mRNA expression

The beneficial effect of BCG in humans, as previously documented in mouse experiments, could be due to an induction of the beneficial Treg cells. The co-evolution of *Mycobacterium* and humans has resulted in *Mycobacterium*-modulated host cell machinery, including de-novo host gene expression by de-methylation of important immune response genes.^31–34^ Chronic infections by *Mycobacterium* evade host recognition on a cellular level by measurable increases in Treg cell numbers and cellular functions.^35^ Treg cells are believed deficient in numbers or function in diverse autoimmune diseases and induction through BCG therapy would be a first step in restoring the immune balance that has been quantified by only flow cytometric methods after BCG.^4,36^ Transcriptional start site (TSS) clusters are located within the Treg-specific demethylation region (TSDR) that is critical for Treg function and that are modulated by de-methylation as was monitored in this study (Supplementary Table S3).

**Fig. 2:**
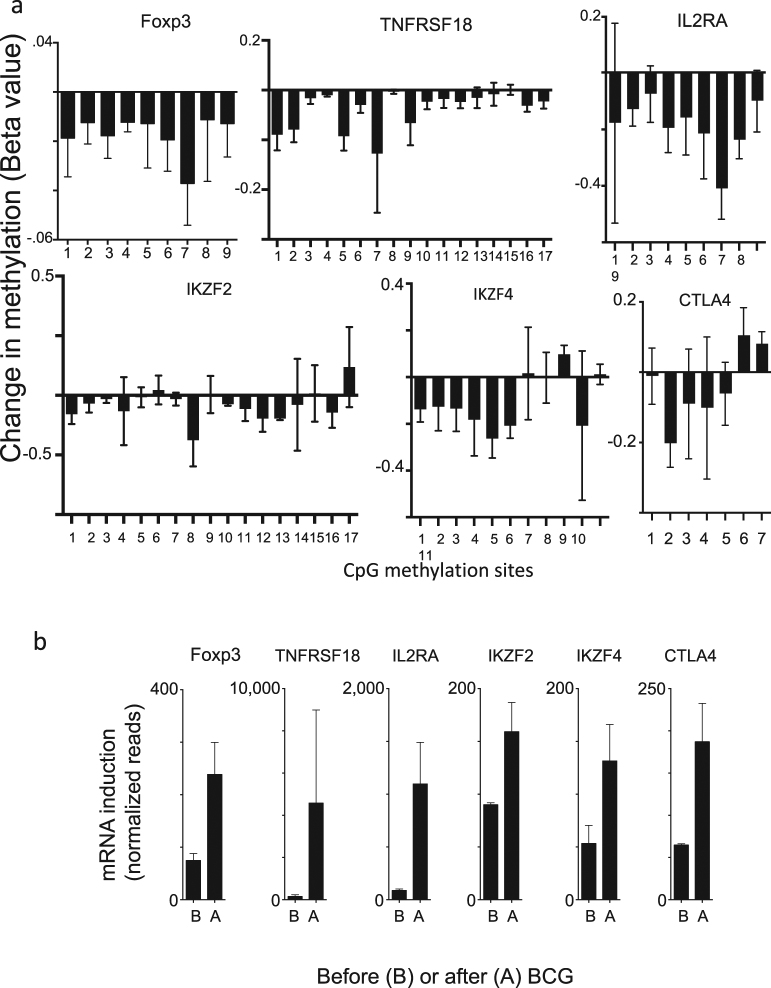
BCG treatment reduces DNA methylation and upregulates expression of Treg signature genes. **a** CD4 T cells were isolated from T1D patients before and after BCG treatment (*n* = 3 subjects; Supplementary Table S1a). DNA was isolated, bisulfite converted and analyzed on the Illumina Infinium HumanMethylation 450 BeadChip array. The data shows that after BCG treatment all six Treg signature genes are demethylated at multiple CpG methylation sites. For a table of the CpG sites used please see Supplementary Table S3. This data compares the methylation state in BCG-treated diabetics 8 weeks after administration of the two BCG vaccines against their pre-treatment baseline. For the Foxp3 gene, all nine methylation sites on the BeadChip were significantly demethylated after BCG treatment (FDR adjusted *p* = 0.004). For the TNFRSF18 gene (also known as GITR receptor), 16 out of 17 methylation sites on the Beadchip were demethylated after BCG and one site was unchanged (FDR adjusted *p* = 0.0008). For the IL2RA gene all 9 methylation sites on the chip showed decreases in methylation after BCG treatment (FDR adjusted *p* = 0.003). For the IKZF2 gene, also known as IKAROS family zinc finger 2 (Helios), there are 17 sites on the chip. After BCG treatment, 13 of those sites were de-methylated, 1 site showed augmented methylation, and 3 sites were unchanged. Overall, demethylation of the IKZF2 sites after BCG treatment was not statistically significant with FDR adjusted *p* = 0.106. For the IKZF4 gene, also known as IKAROS family zinc finger 4 (Eos), there are 11 methylation sites represented on the chip. After BCG treatment, 8 sites were de-methylated and 3 sites showed augmented methylation. Overall the IKZF4 sites were significantly demethylated after BCG treatment (FDR adjusted *p* = 0.038). For the CTLA4 gene, there were 7 sites represented on the chip. After BCG treatment, 5 sites were demethylated and 2 sites showed increases in methylation. Overall there was no significant difference in CTLA4 sites before and after BCG treatment (FDR adjusted *p* = 0.509). This data is from 3 subjects receiving BCG therapy (Supplementary Table S1a). **b** RNA was isolated from PBLs of three T1D before and after in vitro culture with BCG for 48 h and analyzed using RNAseq or transcription profiling. BCG treatment caused a sharp increase in the amount of mRNA as expressed by the number of RNAseq reads for each of the six Treg signature genes that promote Treg function and correlated with the de-methylation patterns

BCG’s impact on methylation was studied at various methylation sites of the following Treg signature genes: Foxp3, TNFRSF18, IL2RA, IKZF2, IKZF4 and CTLA4. T1D subjects were studied before and after (at week 8) in vivo BCG dosing (Supplementary Table S1a). CD4 cells were isolated and genomic DNA was prepared, bisulphite-converted, and then analyzed on Illumina Infinium Human Methylation450 BeadChips (Fig. 2a, Supplementary Fig. S1). Results are expressed in Fig. 2a as change in methylation (after BCG treatment minus before BCG treatment) of the multiple methylation target sites for each gene represented on the BeadChip (Mean ± SEM) or as total change in methylation of all targets per gene (Supplementary Fig. S1). After BCG treatment, the majority of the targets of all six signature genes showed demethylation of most of the methylation control sites. For Foxp3, the false discovery rate (FDR) adjusted *p*-value was 0.004; for TNFRSF18, the FDR adjusted *p*-value was 0.0008; for CTLA4, the FDR adjusted *p*-value was >0.51; for IL2RA, the FDR adjusted *p*-value was 0.003; for IKZF2, the FDR adjusted *p*-value was 0.10; for IKZF4, the FDR adjusted *p*-value was 0.04.

To confirm the observed in vivo epigenetic de-methlyation in the many Treg signature genes after BCG administration, we next profiled the ability of BCG to reciprocally turn on mRNA levels of the same genes. Using transcription profiling we confirmed that the BCG-induced demethylation at the gene level did cause increased mRNA expression of the Treg signature genes; the methylation trends inversely correlated with the mRNA expression levels (Fig. 2b). T1D peripheral blood lymphocytes (PBLs) were cultured for 48 h. with and without BCG. For comparison, averaged data across all methylation sites per Treg signature gene also inversely correlated with increased mRNA for the gene products (Supplementary Fig S1).

### A novel mechanism for treatment of hyperglycemia: induction of the early steps of aerobic glycolysis

Two clinical methods are typically used for improved blood sugar control: increased insulin delivery or decreased peripheral insulin resistance. Because long-term monitoring after BCG treatment failed to show a substantial increase in pancreatic insulin secretion (Fig. 1c), we sought evidence for BCG-induced change in peripheral insulin resistance equivalent to the commercially available Quantose™ IR test (Metabolon, Morrisville, NC). BCG-treated diabetic samples were studied before BCG and up until 4 years after BCG treatment looking for a decrease in insulin resistance. (Supplementary Table S1b; 8 year long followed subjects). In the commercially available Quantose IR test, four metabolites were quantified that are indicative of changes in insulin resistance: alpha-hydroxybutyrate (α-HB), also named 2-hydroxybutyrate, 1 and 2-linoleoyl-glycerophospocholine (L-GPC), and oleic acid.^37^ We therefore analyzed our metabolomics data for changes in these 4 insulin resistance markers. Lowered α-HB is associated with decreased insulin resistance. In our subjects average pre-BCG values were not significantly different from post-BCG values at any time during weeks 4 through year 04 after BCG vaccinations (1.14 ± 0.08 vs. 1.09 ± 0.10; *p* = 0.34). L-GPC is negatively correlated with insulin resistance and impaired glucose tolerance. Pre-treatment diabetic values for 1-LGPC and 2-LGPC were 1.03 ± 0.05 and 1.07 ± 0.05, respectively, whereas post-BCG treatment values were 1.120 ± 0.07 and 1.14 ± 0.07 (*p* = 0.16 and 0.20). Oleic acid is positively correlated with increased lipolysis and insulin resistance. The average pre-BCG value was 1.18 ± 0.10 and post-BCG was 1.38 ± 0.16 (*p* = 0.15). Since the differences for all 4 metabolites were thus not significant, it was unlikely that decreased insulin resistance defined at least from this screening test could explain the reduction in HbA1c due to BCG vaccinations.

**Fig. 3:**
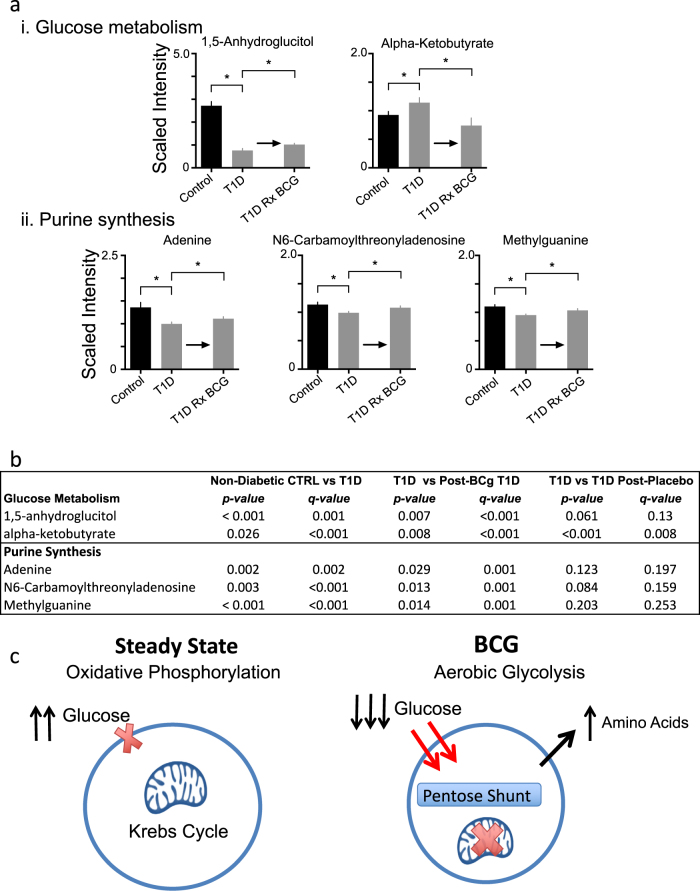
BCG treatment switches cellular metabolism from oxidative phosphorylation to early aerobic glycolysis. **a**, **b** Metabolomic comparisons of the relative levels of intermediates of glucose metabolism and purine synthesis for non-diabetic controls, untreated T1D subjects and T1D patients after treatment with BCG or placebo (sampled biweekly from week 7 to 20 and then yearly through year 5). The results indicate that glucose metabolism is shifted towards aerobic glycolysis in the BCG treated T1D. Asterisks indicate statistically significant differences, which are listed in Fig. 3b. For all metabolomics data, we used an unpaired one-tail Student’s *t*-test that was then corrected for the multiple comparisons with *p* and *q* values. *p* values are given in Fig. 3b. Note that *q* values maintained significance for the T1D results for the BCG treated cohorts. **c** The systemic lowering of blood sugars in T1Ds after BCG vaccines combined with the increased glucose uptake and purine synthesis is consistent with BCG switching cellular metabolism to early aerobic glycolysis. This hypothesis holds that BCG causes downregulation of the Krebs cycle, accelerated aerobic glycolysis, increased glucose uptake, and shunting of glucose to the Pentose Phosphate Shunt for augmented purine biosynthesis

We then investigated a full panel of serum metabolites in BCG-treated and Placebo T1Ds (*n* = 6; sampled bi-weekly from 7 to 20 weeks and then yearly through year 05 compared to untreated T1D diabetic and non-diabetic control subjects (Supplementary Table S1b,c; 8 year long followed subjects). We found two categories of metabolites significantly altered by BCG treatment as compared to the placebo group: glucose processing metabolites and pathway intermediates for de novo purine synthesis (Fig. 3a, b). 1,5-Anhydroglucitol, a sugar metabolite known to increase with lower blood sugar, was lower in untreated T1Ds compared to non-diabetic controls (*p* < 0.001, *q* = 0.001). After BCG treatment it showed a significant increase compared to untreated T1Ds (Fig. 3a, b, *p* = 0.007, *q* < 0.001). Sugar metabolite alpha-ketobutyrate is known to be higher in T1D as compared to non-diabetic controls; we observed this as well (*p* = .026, *q* < 0.001). After BCG treatment alpha-ketobutyrate levels were even slightly lower than control suggesting a meaningful lowering of blood sugars in those cohorts (*p* = 0.008, *q* < 0.001). To demonstrate that the changes after BCG in glucose metabolism and purine synthesis were likely due to the BCG vaccine, the same comparisons of metabolites from the placebo T1D compared to untreated T1D yielded no statistically significant trends toward corrections thus correlating the specificity of the metabolic shifts to BCG vaccinations (Fig. 3b). The T1D to T1D post-placebo comparison for alpha-ketobutyrate yielded a significant *p* and *q* value but this was due to the placebo group having a very low and uncorrected level of this metabolite. While the age of the untreated T1D was slightly shorter (34+/−2 years) compared to the untreated nondiabetic controls (39+/−2 years), all other clinical parameters of age of onset, duration of diabetes and age were closely matched for the BCG-treated T1D subjects compared to the T1D placebo subjects (Supplementary Table S1c).

Three purine intermediates measured by mass spectrometry also showed changes after BCG treatment (Fig. 3a, b, Supplementary Table S1c). Adenine was lower in untreated T1Ds compared to controls (*p* = 0.002, *q* = 0.002). After BCG treatment, adenine levels were closer to normal and were significantly higher than untreated T1D levels (*p* = 0.029, *q* = 0.001). The same trends were observed for two additional purine synthesis intermediates: N6-carbamoylthreonyladenosine (*p* = 0.003, *q* = 0.0005) and methylguanine (*p* < 0.001, *q* = 0.0006) that were both lower in untreated T1D compared to controls. After BCG treatment, N6-carbamoylthreonyladenosine levels were closer to normal and were significantly higher than untreated T1D levels (*p* = 0.013, q = 0.001). After BCG treatment, methylguanine levels were closer to normal and were significantly higher than untreated T1D levels (*p* = 0.014, *q* = 0.001). The same comparisons were done at the same monitoring time points for the simultaneously studied placebo subjects and no significant changes in purines were observed (Fig. 3b; Supplementary Table S4).

The finding that BCG treatment improved glucose metabolism and augmented purine biosynthesis suggests a novel mechanism to explain the BCG-induced reduction of HbA1c: a cellular switch from primarily oxidative phosphorylation, a low glucose utilization state, to augmented early aerobic glycolysis, a high glucose utilization state associated with high purine metabolism (Fig. 3c). It is known that aerobic glycolysis is active at the site of many local infections with low oxygen, including tuberculosis, but not on a systemic level. Indeed the local environment of infections often have augmented glucose utilization and enhanced purine synthesis.^38^ Aerobic glycolysis fuels a dramatic uptake of glucose through regulated cell surface glucose transport and secondary utilization of the pentose phosphate shunt for increased purine synthesis. Our data enabled us to test the hypothesis that the apparently long-term lowering of blood sugars after BCG, even in advanced diabetes, was due to BCG’s systemic induction of aerobic glycolysis and switch from an overactive oxidative phosphorylation metabolism (Fig. 3c).

To help demonstrate that the BCG vaccine was inducing a state of augmented aerobic glycolysis resulting in higher systemic glucose utilization, we examined PBLs and monocyte cultures at baseline and after brief BCG exposures (48 h) for induction of Hypoxia-Inducible Factor 1 Alpha subunit (HIF1A). HIF1A is a master transcription factor that regulates the conversion to high aerobic glycolysis states.^39^ We found that, after brief BCG exposures in vitro, HIF1A mRNA was rapidly upregulated in both PBLs and monocytes of both T1Ds and controls compared to baseline (Supplementary Fig. S2). While this was not a functional outcome, the findings supported our hypothesis of a BCG-mediated switch to augmented aerobic glycolysis on a systemic level similar to what has been observed at local infection sites in the lungs for tuberculosis.^40^

**Fig. 4:**
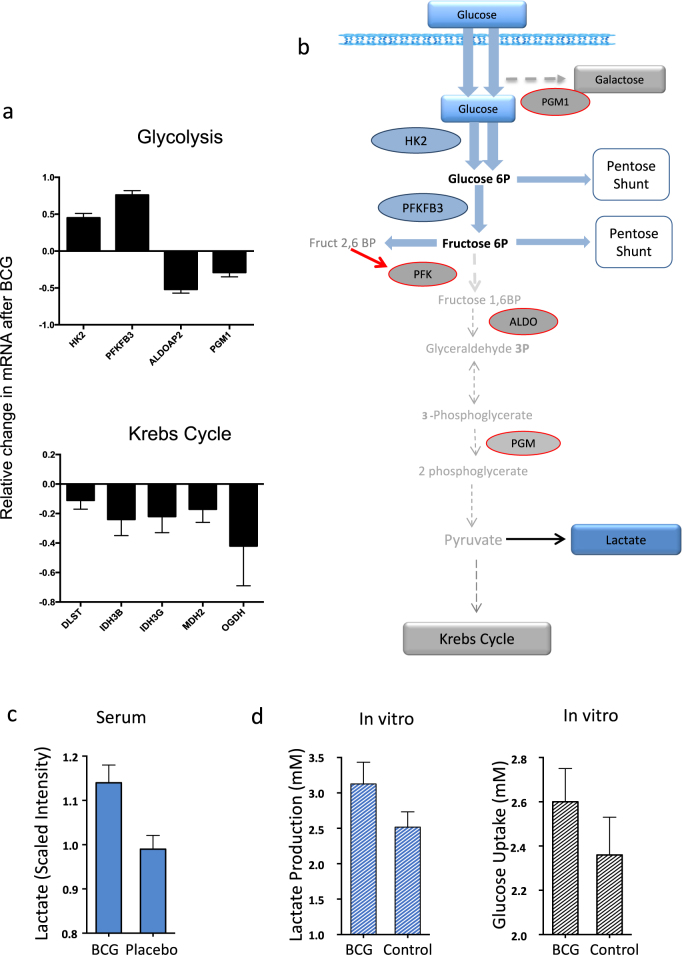
Analysis of mRNA expression and of Metabolites corroborates with the switch from the Krebs Cycle to augmented early aerobic glycolysis after BCG. **a** mRNA expression analysis of type 1 diabetic PBLs before and after BCG treatment in vitro. BCG causes upregulation of mRNA for early glycolysis (HK2, PFKB3), downregulation for late glycolysis (ALDOAP2, PGM1), and a strong downregulation of mRNA for late Krebs cycle steps (bottom). *p* and *q* values for the genes shown are HK2 (*p* = 0.049, *q* = 0.017), PFKB3 (*p* = 0.016, *q* = 0.017), ALDOAP2 (*p* = 0.113, *q* = 0.017), PGM1 (*p* = 0.022, *q* = 0.017), DLST (*p* = 0.074, *q* = 0.017), IDH3B (*p* = 0.084, *q* = 0.017), IDH3G (*p* = 0.084, *q* = 0.017), MDH2 (*p* = 0.090, *q* = 0.017) and OGDH (*p* = 0.070, *q* = 0.017). Combined p values for the Krebs cycle genes using Fisher’s method is 0.005. **b** The schematic summarizes the normal pathway for glucose oxidation via the Krebs cycle and the connecting nodes to the Pentose Phosphate Shunt. Blue rectangles and blue ovals represent upregulated metabolites and mRNA, respectively, after BCG. Gray rectangles and gray ovals represent downregulated metabolites and mRNA, respectively, after BCG. Three BCG-treated T1D subjects were studied. Bars on Fig. 4a, c, d depict SD. The blue shading in Fig. 4b represents the upregulated mRNAs (ovals) and metabolites (rectangles). **c** Serum lactate levels in Phase 1 BCG-treated vs. placebo patients as determined by Metabolon’s GC/HPLC and MassSpec platform one year after treatment. Lactate levels were significantly higher in the BCG-treated patients than placebo-treated patients (*p* = 0.001 and *q* = 0.003) (data from subjects represented in Supplementary Table S1e). **d** Cultured CD4 cells from T1D subjects in the presence or absence of BCG for 48 h (*n* = 25 paired samples) showed both augmented lactate production and accelerated glucose uptake. A short 4-hour collection time of media from either glucose uptake or for lactate secretion after 48 h of BCG or media exposures (control) showed significantly more lactate production after BCG exposures (*p* = 0.025, *n* = 25) and accelerated glucose consumption (*p* = 0.02, *n* = 27) as compared to control lymphocytes that were cultured without BCG

Further corroboration of the concept that BCG treatment switches cellular metabolism towards aerobic glycolysis came from transcription profiling studies (mRNAseq) performed on freshly isolated lymphocytes from T1Ds after BCG treatment. Figure 4b shows the sequential steps of the pathway of glucose metabolism from transport across the cell membrane, early glycolysis, late glycolysis and finally entry into the Krebs cycle both as it relates to enzymes and to metabolites. We examined mRNA for alterations in key enzymes of glucose metabolism in vitro and also for corresponding changes for in vivo metabolites after BCG vaccinations (Fig. 4).

T1D PBLs (*n* = 6 samples; 3 paired samples before vs. after in vitro BCG) were isolated and cultured in the presence of BCG in culture for 48 h. Cells were collected before and after BCG and analyzed by transcription profiling. BCG upregulates expression of early glucose transporters and enzymes involved in early glycolysis (HK2, PFKFB3) (Fig. 4a). BCG downregulates enyzmes involved in late glycolysis (ALDOAP2, PGM1) (Fig. 4a). Consistent with a switch from oxidative phosphorylation to high aerobic glycolysis, Krebs cycle enzymes were also down-regulated (DLST, IDH3B, IDH3G, MDH2, OGDH) (overall Fisher’s *p*-value of 0.005) (Fig. 4a). This data supports a BCG-driven process of a shift in energy metabolism. To further confirm this shift to accelerated glucose utilization through aerobic glycolysis, we studied in vivo serum lactate production and in vitro cellular lactate production and glucose uptake rates after BCG exposures to cultured lymphocytes. Serum lactate levels in Phase I BCG-treated subjects (*n* = 3) vs. untreated T1D (*n* = 50) was determined with mass spectrometry analysis up to the one year time point compared to baseline values. The post-treatment lactate levels rose more in BCG-treated subjects as compared to T1D subjects that received placebo vaccines (*p* = 0.001) (Fig. 4c). This metabolic switch after BCG to aerobic glycolysis could also be observed in vitro (Fig. 4d). T1D PBLs incubated with BCG for 48 h followed by a fresh 4-h collection of media again demonstrated more lactate production (*p* = 0.025, *n* = 25 paired samples) and also accelerated glucose uptake even with even a short 4 h monitoring time (*p* = 0.02, *n* = 27 paired samples). Taken together these results suggest BCG both in vitro and in vivo activated early steps in glucose transport and downregulated the Krebs cycle. This creates in BCG-treated T1Ds an increase in serum lactate. In culture with BCG, lactate was higher as well as the rapid increase in glucose uptake into the T1D lymphoid cells.

**Fig. 5:**
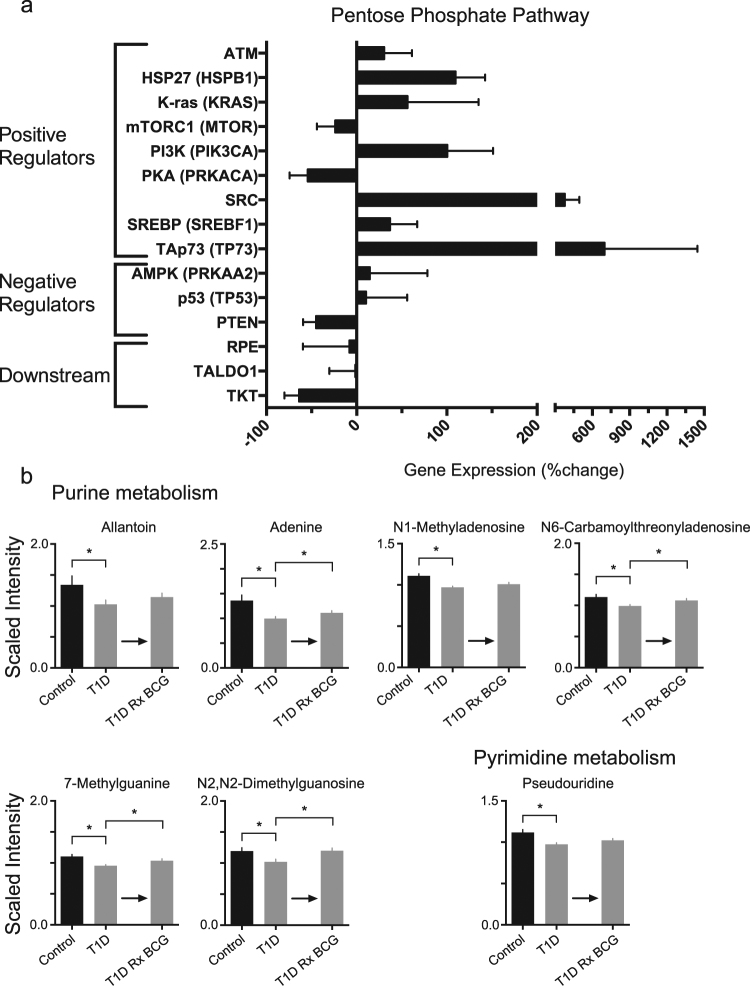
Effect of BCG on induction of the Pentose Phosphate shunt pathway and resulting purine and pyrimidine synthesis. **a** mRNA expression analysis of T1D PBLs treated in vitro with BCG. The graph shows the percent change in gene expression before vs. after in vitro treatment with BCG for 48 h. Positive regulators are mostly upregulated, whereas negative regulators and downstream mediators are mostly downregulated. Three BCG-treated T1D subjects were studied. Bars on the Fig. 5 represent means + /− SEM. Fisher’s Method combined *p*-value for the positive regulators is 0.005. The *p*-values for PTEN in the negative regulators and for TKT in the downstream regulators are almost significant at 0.06 and 0.08, respectively. **b** In vivo metabolomics data of BCG-treated T1D supports the BCG-induced utilization of the Pentose Phosphate Shunt and shows that purine and pyrimidine metabolism are upregulated after BCG treatment. This is consistent with accelerated pentose shunt utilization. Data shows metabolite levels in serum from CTRL (non-diabetic controls) (*n* = 25), T1D subjects (*n* = 50) and post-BCG T1D (*n* = 3 subjects after treatment with two BCG vaccinations). The asterisks represents statistical differences of <0.05; actual statistical values for both *p* and *q* values are given in Supplementary Table S4

A consequence of elevated or restored early aerobic glycolysis should be greater utilization of the pentose phosphate shunt (Fig. 5a, Supplementary Fig. S3). The pentose phosphate shunt yields increased synthesis of purine and pyrimidine metabolites.^41,42^ Our transcription profiling of 48-hour BCG-treated PBLs from T1D subjects showed upregulation of the pentose phosphate shunt consistent with the in vivo metabolomics data (Fig. 5a). There are changes in gene expression for positive regulators and negative regulators of the pentose phosphate shunt, as well as downstream enzymes that divert the pentose phosphate pathway away from purines. A schematic of this pathway and connecting nodes is provided (Supplementary Fig. S3). Seven positive pentose phosphate shunt protein regulators were upregulated (ATM, HSP27, PI3K, SRC, SREBP, K-ras, and TAp73) after BCG treatment of T1D lymphocytes. Fisher’s method combined *p*-value for the positive regulators was 0.005. For example, TAp73 was upregulated by more than 650%. Increases in expression of positive regulators suggest increased throughput of glucose via the pentose phosphate pathway. In contrast, gene expression for negative regulators, such as PTEN of the pentose phosphate shunt, was almost two fold lower (*p* = 0.06), and the downstream pentose phosphate shunt also appeared to be downregulated, consistent with a switch to aerobic glycolysis and diversion towards purine pathways.

We studied serum metabolites of BCG-treated T1Ds to confirm the mRNA expression findings of augmented pentose phosphate shunt utilization and increased purine and pyrimidine metabolites (Fig. 5b, Supplementary Table S1c). Non-diabetic control subjects have significantly higher serum levels of purine and pyrimidine intermediates than untreated TIDs (Fig. 5b, Supplementary Table S4). After two vaccinations of BCG in T1D subjects almost all purine intermediates increased. In the purine pathway, adenine, N6-carbamoylthreonyladenosine, 7-methylguanine and N2,N2-dimethylguanosine all statistically showed significant increases in BCG-treated T1Ds compared to untreated T1Ds (*p* = 0.029, *q* = 0.001; *p* = 0.013, *q* = < 0.001; *p* = 0.014, *q* = < 0.001; *p* = 0.002, *q* = <0.001, respectively). Allantoin and N1-methyladenosine also rose in BCG-treated T1Ds, but not significantly. Pseudouridine, a member of the pyrimidine metabolic pathway, was significantly lower in the untreated T1D group as compared to the non-diabetic controls, again suggesting underutilization of early aerobic glycolysis (*p* < 0.001, *q* = 0.001). After BCG, pseudouridine showed a small increase in BCG-treated as compared to untreated T1D that approached statistically significance (*p* = 0.057, *q* = 0.002). *p* and *q* values for all comparisons are listed in Supplementary Table S4. The BCG-induced switch to high glucose transport and shunting to the pentose phosphate pathway is illustrated in Supplementary Fig. S3 as it relates to the monitored proteins (ovals) and metabolites (rectangles) of this pathway.

### BCG-induced aerobic glycolysis: implications for all forms of hyperglycemia

We next tested the hypothesis that BCG should be able to lower blood sugars regardless of the cause of hyperglycemia. The data presented above suggests a novel way to systemically regulate blood sugars, independent of insulin. This novel approach for blood sugar regulation appears to hinge on a systemic switch from primarily oxidative phosphylation to early aerobic glycolysis resulting in the acceleration of glucose uptake. If this novel mechanism of systemic blood sugar control is driving the return towards normoglycemia after BCG treatment, the data would suggest that this mechanism is independent of the underlying etiology of hyperglycemia—in this case autoimmune type 1 diabetes. Autoimmunity was not essential for BCG to lower blood sugars.

**Fig. 6:**
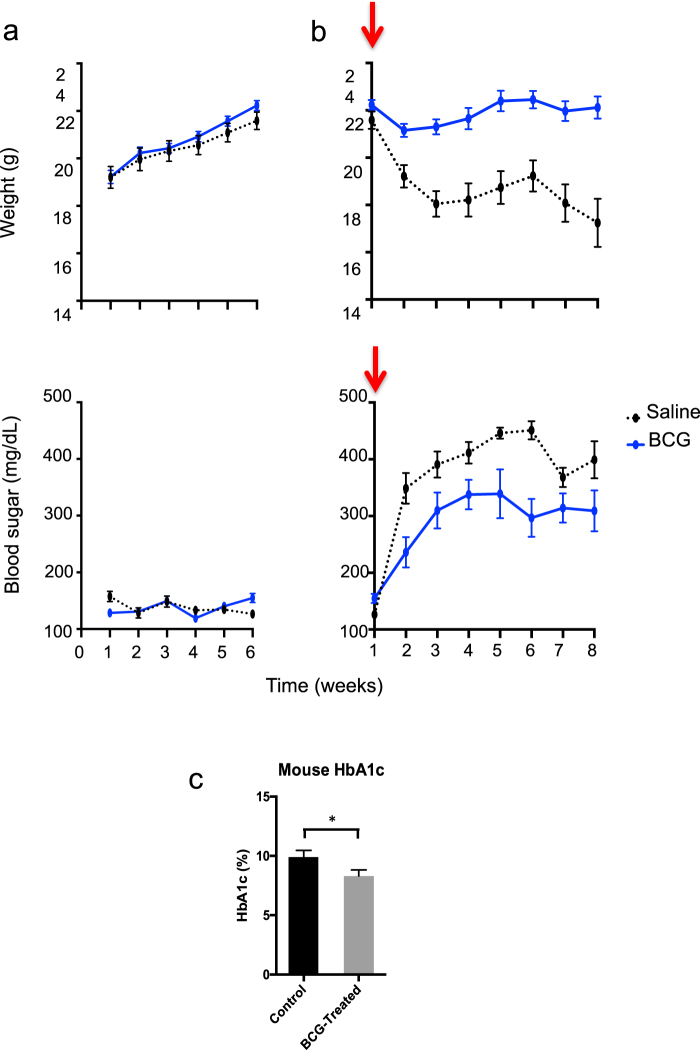
BCG pre-administration reduces hyperglycemia in chemically-induced (Streptozocin) mice but does not induce hypoglycemia in normal mice. **a** Normal BALB/c mice were first studied in a normoglycemic state with (*n* = 12) and without BCG (*n* = 12) treatment for blood sugars and weight (left panels). BALB/c mice were rendered chemically diabetic (arrows) and studied with and without BCG treatment six weeks earlier with preventative pre-injections (right panels). Most mice became severely hyperglycemic after treatment with streptozocin (STZ) which selectively kills the insulin-secreting cells in the pancreas. All mice were monitored for blood sugar levels and weighed on a weekly basis. BCG-treated mice gained weight at the same rate as untreated control mice and had normal blood sugars with no indication of hypoglycemia (left panels, blue lines). After STZ induction of hyperglycemia, the control mice rapidly started to lose weight and became severely hyperglycemic within one week (right, black lines). In contrast, mice first treated with BCG before STZ treatment were able to maintain their weight and had markedly lower levels of hyperglycemia (right, blue line). **b** Measurements of HbA1c values in STZ-treated BALB/c mice after 6 weeks with and without prior BCG treatment show the protection afforded by BCG and the resulting lower HbA1c values of 85 ± 6.6 mmol/mol (9.9 ± 0.6% NGSP) without BCG vs. 67 ± 5.5 mmol/mol (8.3 ± 0.5% NGSP) with BCG treatment; *p* = 0.02, *n* = 19 surviving mice). **c** At 8 weeks after the induction of hyperglycemia, the BCG-treated mice had statistically lowered HbA1c values

To test the hypothesis that BCG in vivo can induce a switch to systemic aerobic glycolysis with clinical significance, i.e., sufficient to lower blood sugars, we turned to murine testing. We used Streptozotocin (STZ) as a chemical that selectively destroys the insulin secreting islet β-cells in the pancreas to induce hyperglycemia as a way to elevate blood sugars and test for glucose utilization. We wanted to determine the impact of BCG vaccinations in normal mice with and without hyperglycemia and in the absence of autoimmunity. Normal C57BL/6 mice (*n* = 24) were divided into two groups. One group (*n* = 12) was treated with 0.1 mg BCG whereas the other group (*n* = 12) remained untreated. For this series of experiments all the mice remained normoglycemic. Weight and blood sugars were determined weekly for 6 weeks. Figure 6a (upper left) shows that BCG-treated mice gained weight at the same rate as controls. Likewise, blood sugars in the BCG-treated mice were indistinguishable from controls (Fig. 6a, lower left). Thus BCG alone affected neither weight nor glycemic control in healthy mice.

We tested the effects of hyperglycemia induction with STZ when one group of mice received BCG (*n* = 12) and one group of mice received saline (*n* = 12) six weeks earlier (Fig. 6b). Control mice given only STZ rapidly started to lose weight (Fig. 6b, upper right) and became severely hyperglycemic (lower right). In contrast, the BCG-treated mice maintained their weight at a constant level (upper right; *p* < .0001) and their blood sugars did not rise as high as the controls and plateaued at a much lower level (Fig. 6b, lower right; *p* = 0.002). At week 8 after STZ treatment, the BCG-treated mice had a significantly lower HbA1C as compared to the control mice (Fig. 6b, *p* = 0.02). It should be noted, that the at least 6 week dosing lag in the administration of BCG prior to a glucose challenge was obligatory to see the maximal protective effect of improved blood sugar control. BCG can thus significantly lower blood sugars without underlying autoimmunity, and BCG has no deleterious effect by lowering blood sugars lower than normal. BCG treatment does not carry the risk of hypoglycemia as is the case for intense insulin therapy.

## Discussion

Our 8-year long clinical trial of monitoring subjects receiving *Mycobacterium* re-introduction through the BCG vaccine triggers two clinical effects in humans with established T1D: stable and long-term reductions in blood sugar and epigenetic changes in Treg signature genes for restored tolerance. Both beneficial effects appear to be driven by a systemic metabolic shift from oxidative phosphorylation towards accelerated and early aerobic glycolysis. The significant clinical effects, using BCG with intradermal dosing, took three years to occur but then held steady for at least five additional years without further interventions.

Aerobic glycolysis is a metabolic state with high cellular glucose utilization and rapid ATP production.^38^ This beneficial metabolic switch after BCG administration in vivo is detected by diverse methodologies including transcription profiling, epigenetics, metabolomics and clinical monitoring. The BCG-specific effect on metabolism included increases in key early glycolytic enzymes, upregulation of glucose uptake with systemic lowering of blood sugars, shunting the accelerated glucose utilization through the pentose phosphate pathway, decreased utilization of the late glycolytic steps including the Krebs cycle, increased lactate production and decreased oxidative phosphorylation. Taken together, the findings suggest a novel way to regulate blood sugars levels using attenuated *Mycobacterium bovis*.

BCG also demethylated Treg signature genes. Treg-specific DNA hypomethylation is central in gene upregulation for Treg function.^43^ The demethylation of Treg signature genes also correlated with increased mRNA expression of the genes involved in the de-methylation process.^43^ Published studies confirm that Treg-specific DNA hypomethlylation is instrumental in gene upregulation in steady state Tregs. *Mycobacterium* especially in the form of tuberculosis and leprosy are known to create granulomas encircled with T-regulatory cells.^33^ BCG induces a similar systemic induction of Treg cells^4^ and supplements the decrease in systemic inflammation that also drives type 1 diabetes inflammation.^44^ Epigenetic reprogramming of the host by diverse infections, including *Mycobacterium tuberculosis*, or beta-glycan, a component of *Candida albicans* occurs at the levels of key checkpoints, including methyltransferases and key steps in aerobic glycolysis such as HIF1α.^45–48^ Here, we observed these same immune and metabolic changes as it relates to genes involved in the reestablishment of tolerance and glucose utilization in humans in vivo in response to BCG vaccinations. The changes in receptor controlled glucose utilization in this case are therapeutically useful for blood sugar lowering in type 1 diabetes.

An induced metabolic shift in diabetic human subjects toward aerobic glycolysis, resulting in accelerated glucose utilization represents another advantage of this novel metabolic method to regulate blood sugars. Although this is a cellular mechanism to increase glucose utilization, no reported cases of severe hypoglycemia occurred in BCG vaccination subjects during 5 years of monitoring after the HbA1Cs returned to the near normal range. Clinical trials have shown similar, albeit less uniform, lowered blood sugar with introduction of intense insulin treatments combined with intense monitoring, insulin pumps, glucose sensing equipment, diet, exercise and frequent health care supervision. Such intense treatments come with greatly increased risk of hypoglycemic episodes with morbidity and mortality. In general, a clinical goal of a HbA1c near 7.0 mg% is set to prevent the lethal effects of hypoglycemia from the lowering of blood sugars by conventional insulin therapy. Lowering of hyperglycemia, measured with HbA1c measurements, is associated with clinically significant declines in death, eye disease, kidney disease, nervous system disease and microvascular and macrovascular disease.^49,50^ For each 10% change or 11 mmol/mol (1% NGSP, National Glucose Standardization Program) reduction in absolute HbA1c values there is a relative risk reduction of 21% in diabetes outcomes, 21% in diabetes deaths, and 37% in microvascular complications. We now introduce a simple and inexpensive way to achieve long lasting lowered blood sugars without hypoglycemia, even in long-standing disease. We achieve these lowered blood sugars uniformly in all treated subjects for over 5 years with no additional health care costs or subject compliance requirements. To confirm the systemic benefits of aerobic glycolysis we modeled the impact of administering BCG to normal mice. Both treated and untreated mice had normal blood sugars, demonstrating, that unlike insulin, the BCG-induced metabolic shift to aerobic glycolysis does not induce hypoglycemia and the increased glucose utilization by BCG is receptor regulated. Importantly when BCG-treated normal mice were made diabetic with STZ, a chemical that destroys pancreatic insulin-secreting cells, blood sugar reductions of at least 30% were achieved. This suggests BCG induction of aerobic glycolysis is naïve to the underlying cause of the elevated blood sugars and might have broader applicability to other forms of hyperglycemia like type 2 diabetes.

Alterations in immune metabolism are known to be closely linked to infections including *Mycobacterium tuberculosis*. In the infected granulomas of murine lung tissue, there is up regulation of key glycolysis enzymes and transporters for glucose uptake, down regulation of enzymes participating in the Krebs cycle and downregulated oxidative phosphorylation.^51^ Tuberculosis, similar to the systemic BCG vaccines described here, induces hypoxia inducible factor 1 alpha (HIF-1a), a key enzyme in aerobic glycolysis control. BCG also changed glycolysis in vitro with increased glucose uptake of infected monocytes, a process similar but not identical to the Warburg effect.^40,52^ Before this 8-year long trial, it was not appreciated that infection with *mycobacterium* as a avirulent strain could have such a profound and permanent therapeutic effect on a systemic basis.

Why does BCG’s reduction of HbA1c in humans take 3–4 years? This crucial question cannot be directly answered by our clinical trial. We can speculate that since autoimmune disease takes years to develop, the reversal or halt of autoimmunity may share similar time dependence. It is important to note that the 3-year lag time was also found in a human clinical trial of BCG’s benefits for multiple sclerosis.^12^ In our study of STZ-treated mice, a lag time of at least 6 weeks was needed to show a reduction in HbA1c, which, given the 2.5 year lifespan of a mouse, translates to approximately 3.5 years in humans. Since BCG is a live vaccine, it is assumed that BCG’s effects have to become systemic for its correction of HbA1c. It is speculated that the first signs of a Treg induction effect at six weeks after two vaccinations may need to become systemic. The shift to aerobic glycolysis may involve additional cells and organs beyond what was examined here. More research is needed to understand the kinetics of BCG’s effects, including whether more frequent dosing of BCG can shorten the lag time and if the systemic delay but durability of the clinical response is related to co-infection of stem cells that then create an entire immune reservoir for blood sugar regulation.

The health benefits of the BCG vaccine reported here suggest reconsideration of vaccine policy in areas of the world without endemic tuberculosis. Not one, but multiple, BCG doses are necessary to prevent T1D, based on epidemiological and animal studies.^10,25^ Studies have already reported that the BCG vaccine alters the susceptibility to infections with unrelated organisms at later times during life. Animals injected with *Mycobacterium bovis* as the BCG vaccine demonstrate acquired resistance to *Stapylococcus aureus, Herpes virus, Salmonella, Listeria monocytogenes and Candida albicans*.^53–55^ This has also been observed in humans since 1928. Researchers at the Institute Pasteur observed that the BCG vaccine conferred mortality protection, and additional human studies continue to observe this benefit on a global level.^54,55^ We are only beginning to appreciate the evolutional synergy of the reintroduction of the *Mycobacterium bovis* attenuated BCG organism into modern day humans.

In conclusion, we show that re-introduced avirulent *Mycobacterium bovis* in the form of BCG vaccine holds benefits for human disease blood sugar control and re-establishment of tolerance through Treg cells. Although this study is centered on T1D this novel mechanism might be speculated to also be applicable to T2D. The limitations of this therapy as it is currently formatted as intradermal vaccinations are the almost 3+ year lag time for a systemic effect. Also to prove efficacy in the broadest T1D subject populations, including pediatric subjects, there is now the need to expand the trial to larger number of subjects of all ages and durations of diabetes. The advantages of this therapy are its durability, simplicity, safety, and cost-effectiveness. Exploitation of safe versions of *Mycobacterium* for systemic changes in immune-metabolism may recapitulate the immense benefit of co-evolution of the virulent genetic organisms, genomic modeling by epigenetics and restoration of a beneficial host partnership in modern societies. Perhaps attenuated *Mycobacterium* are primary organisms for reestablishing host-environment interactions for improved health.

## Materials and methods

### Clinical trial and research study participants

This is a study of 282 human research participants for both in vivo BCG vaccine clinical trial studies (*n* = 52) and in vitro mechanistic studies (*n* = 230). Of these total research subjects 211 had type 1 diabetes and 71 were non-diabetic control subjects.

The in vivo study of BCG vaccinations is comprised of adult T1D subjects receiving BCG, receiving placebo vaccinations and simultaneously studied reference type 1 diabetic subjects followed with the same standard of care (Supplementary Table S1a, b). For all BCG treated subjects followed for up to 5 years, in total of 52 subjects, their clinical traits are represented in Supplementary Table S1a. Some BCG and placebo treated Type 1 diabetic subjects were enrolled and were studied for 8 years (Supplemental Table S1b, *n* = 6 subjects); other BCG treated (*n* = 9), placebo treated T1D (*n* = 3) and reference T1D (*n* = 40) were studied for up to 5 years (Supplemental Table S1a). Eight year-long studied subjects all participated in the Phase I BCG clinical trial of 20-weeks duration (Study #2007P001347; NCT00607230); all participated in this Phase I BCG long-term clinical follow-up study for up to 8-years duration after study start (Study #2012P002243). Details of the Phase I BCG clinical trial have been previously published after 20 weeks of observation.^4^ All other BCG treated T1D subjects with under 5 years of participation were subgroups of subsequent BCG clinical trial studies (Study #2013P002633).

T1D and control subjects in this study also included volunteers who donated blood for in vitro studies under Study #2001P001379 and were compared to the in vivo studied subjects receiving BCG or placebo vaccination or compared to in vitro studied subjects. Supplementary Table S1c are the clinical traits of the 106-untreated type 1 diabetic subjects and 50 non-diabetic control subjected used in the metabolonic studies for comparison to the in vivo treated BCG or Placebo subjects. Supplementary Table S1d are the clinical traits of the type 1 diabetes subjects used in the RNAseq studies to understand the change in lymphocyte gene expression after in vitro BCG exposures (*n* = 3). Supplementary Table S1e are the clinical traits of T1D (*n* = 23) or control non-diabetic subjects (*n* = 10) used to study lactate production in vitro after BCG exposures. The production of lactate after BCG or placebo vaccinations in vivo are represented by the T1D subjects in SupplementaryTable S1b (*n* = 3 BCG treated, *n* = 3 placebo treated). Supplementary Table S1f are T1D (*n* = 27) and non-diabetic control (*n* = 27) subjects used in Fig. 4d, Supplementary Fig. 2 and Supplementary Fig S5c, in vitro studies related to lactate production and glucose uptake, in vitro PBL and monocyte production of HIF1A and NR1H3 induction with BCG. All human studies had full MGH institutional approval through Massachusetts General Hospital and Partners Health Care; the BCG interventional studies were also formerly approved through the FDA IND#2007P001347 and IND#2013P16434.

All subjects provided informed consent. It is important to note that all BCG-dosed subjects, for the duration of this 8-year study, especially during the 3–4 year period where they had a major drop in their HbA1c, had no change in their BMI, no change in their use of associated technologies (i.e., insulin pumps, continuous glucose meters), and no adoption of ketotic diets and we were all in this study blinded to this data until we re-opened the study to examine previously collected HbA1c values.

For this BCG trial, the Connaught strain of BCG was utilized twice and administered intradermally in the right and left upper arm with a syringe 4 weeks apart. Hypoglycemia was defined and monitored by the hypoglycemia survey forms developed by Clark et al.^56^

All human subjects studied in this paper are represented in Supplementary Table S1 with their clinical traits and reference to the manuscript Figure or Table site where the data is presented. Supplementary Table S1 also statistically compares the varies study group for parity as it relates to their age, age of onset (AOO) and duration of type 1 diabetes (two-sided unpaired *t*-test) to show in almost all cases close parity of the clinical traits between contrasting treatment and control groups.

### Mice

BALB/c male mice were purchased from The Jackson Laboratory (Bar Harbor, ME) and housed at five animals per cage at the MGH animal facilities (four animals per cage after they reached a weight of >25 g). Studies had full IACUC approval under protocol #2008N000176 and involved at all times the ethical treatment of animals. We determined HbA1c from mouse blood samples using the A1CNow + kit from PTS Diagnostics (Indianapolis, IN). For mouse blood sugars we used an ACCU-CHEK Aviva blood glucose meter (Roche, Indianapolis, IN). BCG was administered by hind footpad injection (0.10 mg BCG in 25 μL saline in one footpad). Induction of hyperglycemia was performed using a single intraperitoneal injection of Streptozotocin (Sigma-Aldrich, Milwaukee, WI) at 150 mg/kg in PBS.

#### Clinical chemistries

All human HbA1c, glucose levels were processed directly from fresh blood by certified diagnostic laboratories approved by The Massachusetts General Hospital. Human serum samples were assayed for C-peptide using regular (Cat# 10-1136-01) or ultrasensitive (Cat# 10-1141-01) C-peptide ELISA kits from Mercodia AB (Uppsala, Sweden) using −80 ℃ frozen serum as previously described.^57^

#### Intravenous glucagon stimulation test

Subjects were asked to consume at least 150 g of carbohydrates for the 3 days prior to the intravenous glucagon stimulation test (GST) and fast for 8 h the night before. On the morning of the GST, subjects were asked to withhold insulin until after they had completed the test. The GST was only administered when blood sugars were between 3.9 mmol/L (70 mg/dL) and 12.5 mmol/L (225 mg/dL). A 20-22 gauge angiocatheter was placed and a 5 mL blood sample taken for baseline glucose and for baseline C-peptide levels. Six minutes later, 1 mg of glucagon was administered intravenously. An additional 5 mL blood sample was then collected at 6 min. Serum was prepared from the blood samples and frozen at −80 ℃ for subsequent C-peptide Elisa as previously described.^3^

#### Isolation of CD4 T cells

CD4 T cells were isolated from blood from purple tops using magnetic EasySep Direct Human CD4+ T Cell isolation kits from Stemcell Technologies (Vancouver, BC, Canada, Cat# 19662), following the instructions of the manufacturer. Briefly, 1200 μL of Isolation Cocktail and 1200 μL of RapidSpheres were mixed with 24 mL of whole blood in a 50 mL centrifuge tube and incubated for 5 min at room temperature. Twenty-four milliliter of PBS was then added and the tube placed into a “Easy 50” magnet (Stemcell Technologies, Cat# 18001). This immobilized the unwanted non-CD4 cells at the side of the tube. After 10 min, the CD4-enriched cell suspension was then transferred into a new tube and the magnetic separation process repeated for 5 min with fresh Isolation Cocktail and RapidSpheres. The resulting highly CD4 enriched cell suspension was transferred into a new tube and purified for a third time using the magnet. The resulting final CD4 cell preparations had purities of >95%.

#### Methylation analysis

Female BCG-treated T1D subjects were studied at several time points before and after BCG administrations for changes in methylation of their peripheral blood CD4 T cells. These T1D subjects had blood drawn before the first BCG injection on Day 0 and then again at 8 weeks after receiving the second BCG vaccination at week 4. CD4 cells were then isolated and DNA prepared and stored at −80 ℃. For each sample, 1 μg of DNA was bisulfite-converted using the EZ-96 DNA Methylation™ Kit (Shallow Well Format) (Zymo Research Corporation, Irvine, CA) following the manufacturer’s protocol with modifications in incubation conditions as recommended for the Illumina Infinium Methylation Assay. To verify the quality of bisulfite conversion assay, standard PCR of the highly methylated P19 gene was performed using primers targeting known methylation sites. These primers will anneal to these sites only if the bisulfite conversion is successful. The primers for the methylated targets are: Bisulfite_QC.F (CTAAAACCCCAACTACCTAAA) and Bisulfite_QC.R (TAGGTTTTTTAGGAAGGAGAG). PCR cycling conditions are 95 ℃ for 15 min, 5 cycles of 95 ℃ for 30 s, 60 ℃ for 2 min, 72 ℃ for 1 min, 30 cycles of (95 ℃ for 30 s, 65 ℃ for 1 min, 72 ℃ for 1 min), and 72 ℃ for 5 min. PCR products were run on a 2% agarose gel and a single band at 286 bp indicated successful conversion.

The methylation assay was then performed on the Human Methylation450 BeadChip (Illumina, San Diego, CA) using 200–400 ng of bisulfite-converted DNA product for each sample according to the Infinium HD Methylation Assay protocol (Illumina). This assay targets over 450,000 known and potential methylation sites across the genome via 50-mer probes attached to the Infinium BeadChips. The bisulfite converted DNA was hybridized to the probes on the BeadChip followed by a single base extension to incorporate a fluorescent-labeled nucleotide (Cy3 or Cy5) at the target position to differentiate methylation status. The BeadChips were then imaged on the Illumina iScan reader and the methylation level of each CpG locus was calculated in the GenomeStudio® Methylation module as methylation beta-value (β = intensity of the methylated allele (M)/(intensity of the unmethylated allele (U) + intensity of the methylated allele (M) + 100).

#### Transcription profiling analysis (RNAseq)

Total RNA was isolated from peripheral blood lymphocytes using the RNeasy Plus Mini Kit of QIAGEN (QIAGEN Sciences, MD, Cat# 74104) and processed by the CCCB (Center for Cancer Computational Biology) at Dana Farber Cancer Institute (Boston, Mass). RNA quality was determined using the Agilent 2100 bioanalyzer. RNA with a RIN value greater than 6 and less than 10% DNA contamination was used for library preparation. Using an input of 100 ng of total RNA, poly-A selection was performed using a NEBNext® Poly(A) mRNA Magnetic Isolation Module. The resulting mRNA was used for library preparation using the NEBNext® Ultra™ Directional RNA Library Prep Kit for Illumina®. The RNAseq libraries were run on a high sensitivity DNA chip on the Agilent 2100 Bioanalyzer, and the functional concentration of the library was determined through qPCR using the KAPA Biosystems Illumina Library Quantification kit. Libraries to be sequenced were pooled at a concentration of 2 nM, and were denatured and diluted to a final concentration of 2 pM and loaded onto the Illumina NextSeq 500. Alignment of reads against reference genome HG19 was performed using TopHat and analyzed using Cufflinks.

The resulting data was then normalized using DESeq, part of the R—Bioconductor package.^58^ Normalized data was analyzed using the Ingenuity Pathway Analysis software from QIAGEN, the R Statistical Programming language,^59^ and Microsoft Excel.

#### Metabolomic analysis

The metabolomic study compared serum samples from control non-diabetic subjects (*n* = 50) to serum samples from untreated T1Ds (*n* = 106). It also compared the untreated T1Ds (*n* = 50) to post-BCG T1Ds (*n* = 3, sampled biweekly from 7 to 20 weeks and at year 05). All metabolomics study samples were sent for metabolic profiling and statistical analysis to Metabolon Inc (Durham, NC). Serum samples were analyzed on Metabolon’s integrated discovery platform consisting of gas and liquid chromatography for separation and mass spectrometry for detection and identification. This high throughput platform, among other studies, has been used for the identification of biomarkers of insulin resistance in patients at risk for Type 2 diabetes.^60^ This resulted in the development of the Quantose IR test, which measures four metabolites for the assessment of insulin resistance^37^ In our serum samples, the platform was able to distinguish a total of 690 different metabolites.

#### In vitro glucose utilization assay on CD4 cells

CD4 cells were isolated from whole blood using the Direct Human CD4+ T Cell Isolation kit from Stemcell Technologies, (Vancouver, Canada) as per the manufacturer’s instructions. The cells were then incubated in 24 well plates for 48 h at 37 °C at 1 × 10^6^ cells per well in assay medium (1 mL RPMI with Glutamax, 2% FBS, Pen/Strep, 400 Units of IL-2 (Gibco), 25 μL CD3/CD28 (Stemcell Technologies)). Wells were then supplemented with 156 × 10^3^ CFU of BCG. Control wells did not receive BCG. After culture, the cells were washed with PBS and resuspended in fresh assay medium and incubated for 4 h at 37 °C at a concentration of 250,000 cells/50 μL. Following this incubation period, media supernatants were collected and stored at −80 °C. Baseline glucose control media consisted of assay medium that was incubated for 4 h at 37 °C without cells. Supernatants were analyzed using the Glucose Colorimetric Assay Kit (BioVision, Milpitas, CA) as per the manufacturer’s instructions. Samples were diluted 100× then filtered using Amicon Ultra-0.5 Centrifugal filters (15 min at 14,000×*g*) before analysis.

#### Lactate assay

Serum samples and culture supernatants were analyzed using the Lactate Colorimetric Assay Kit II (BioVision, Milpitas, CA) as per the manufacturer’s instructions. Samples were diluted 30× in assay buffer then filtered using Amicon Ultra-0.5 Centrifugal filters (15 min at 14,000 × *g*) before analysis.

#### Statistical methods

Statistical significance for flow cytometer data and metabolomics was determined using the unpaired one-tail Student’s *t*-test. For non-normal distributed data we used the Wilcoxon Signed Rank test for glucagon stimulated C-peptide data and for Treg Signature Genes RNAseq data. Where indicated, combined *p*-values were calculated in R using Fisher’s method, also called Sum of Logs method or Chi-Square (2) method, using the METAP library.^61–63^ Relative (percent) change rate of HbA1c was compared using the linear mixed effects model with subject-level random effects. The change rates in the control, placebo and BCG groups were compared based on the statistical significance of the interaction term between time and group indicator in the linear mixed effects model. For statistical analysis of the methylation data, we calculated the differences between the triplicate T1D treated BCG and untreated T1D samples Beta values for each CG-target of the 6 Treg signature genes (see Supplementary Fig. 3); then for each signature gene calculated mean methylation changes and SEM’s using these differences; and then calculated the T-statistic for each gene, assuming a population average of 0, i.e., no change in methylation in the absence of BCG treatment. The *p*-value was then calculated using the T statistic and adjusted for false discovery rate using a Bonferroni correction. Statistics were performed using SAS version 9.4 (SAS Institute, Inc, Cary, NC), the R Statistical Computing Language,^5^ or in Microsoft Excel. Confidence levels were set to 0.05.

### Data availability

The raw metabolomic and epigenetic data presented in this paper is available upon request.

## Electronic supplementary material

Supplementary Materials
