# Supplementary material for: Long-term reduction in hyperglycemia in advanced type 1 diabetes: the value of induced aerobic glycolysis with BCG vaccinations
Source: NPJ Vaccines. 2018 Jun 21;3:23. doi: 10.1038/s41541-018-0062-8 (PMC6013479; doi:10.1038/s41541-018-0062-8)

Online Content

Supplementary Tables and Figures

Supplementary Table S1 Baseline clinical characteristics of all T1D and control subjects. Sample sizes and clinical characteristics of subjects studied in the clinical trial for up to 5 or 8 years (**a, b**), in the clinical trial for in vivo epigenetic modifications (**a**), for RNAseq (**d**) for in vivo metabolomics studies **(a, b, c),** lactate production (**e**) and general in vitro studies (**f**). Also represented under each subject section (Supplementary Table S1a-f) is a statistical comparison of the clinical traits of age, age of onset (AOO), and diabetes duration to show equivalent of the various study groups. Represented in Supplemental Table S1 is the location of the data throughout the manuscript. Also throughout the manuscript we refer to the subjects in Supplementary Table S1a as “up to 5 year followed subjects” and the subjects in Supplementary Table S1b as “8 year long followed subjects” to add clarity and subject tracking.

Supplementary Table S2 Long-term improvement of glycemic control in T1Ds after BCG treatment. The data shows absolute values and p-values for HbA1cs of T1Ds dosed twice with BCG as compared to both the placebo-T1Ds and the reference-T1Ds of untreated patients receiving the same standard of care. The data cover the 8-year observation period and thus represents the 8 year long followed subjects.

Supplementary Table S3 Target IDs for the Treg signature genes on the Illumina Infinium Human Methlyation 450 BeadChip. These target genetic regions were analyzed on the T1D samples from subjects receiving BCG treatment and compared to untreated methylation patterns before BCG.

Supplementary Table S4. Statistics for metabolites as they relate to purine and pyrimidine metabolism. There were 3 BCG treated T1D compared to 106 untreated T1D and 50 non-diabetic control cohorts.

Supplementary Figure S1 BCG treatment in vivo upregulates mRNA expression (**a**) and reduces DNA methylation of Treg signature genes (**b**). **a** RNA was isolated from PBLs of T1D patients (n=3) before and after in vitro culture with BCG for 48 hrs. and analyzed using mRNA expression profiling (n=6 samples). BCG treatment caused a sharp increase in the amount of mRNA as expressed by the number of RNAseq reads for each of the six Treg signature genes that promote Treg function and correlated with the de-methylation patterns observed in vivo (**b**, see below). (Wilcoxon Signed Rank Test, p=0.031) (top figure). **b** CD4 T cells were isolated from three T1D patients before and after they were treated with BCG *in vivo* (n=3 patients; 6 samples) (bottom figure). DNA was isolated and analyzed on the Illumina Infinium HumanMethylation450 BeadChip array. The data shows that after BCG treatment all six Treg signature genes are de-methylated at multiple CpG methylation sites. This data represents BCG-treated diabetics 8 weeks after the two BCG vaccines compared to pre-treated baseline methylation state. The data bars for each gene represent the average change in mRNA for all methylation sites of each gene. Taken together, the six Treg-signature genes are significantly de-methylated after BCG treatment (p=0.016).

Supplementary Figure S2 The master transcription factor Hypoxia-Inducible Factor 1 Alpha subunit (HIF1A) is a key regulator of the cellular switch from high oxidative phosphorylation to aerobic glycolysis. HIF1A mRNA is upregulated, both in PBLs and in monocytes, after 48 hrs BCG treatment in T1Ds and controls (n=4 T1D for PBLs and monocytes, n=4 controls for PBLs and monocytes).

Supplementary Figure S3 Pentose Phosphate shunt and biosynthesis of Purines and Pyrimidines. The schematic summarizes the metabolic pathway of Pentose Phosphate shunt and the connection to Purine and Pyrimidine pathways. Increased metabolites (rectangles) and mRNA regulators (ovals) after BCG treatment are shown in blue and turned off metabolites (rectangles) and mRNA regulators (ovals) after BCG are shown in white or grey.

Supplementary Table S1

a. Clinical traits of the BCG treated type 1 diabetic, Placebo and Reference type 1 diabetic subjects used to study the 5-year long term in vivo effects of the BCG vaccine (Fig 1a,b,c, Fig 2a, Fig 3, Fig 4a, 4c, Supplementary Fig 1S, Supplementary Table S2, Supplementary Table 4S)(Subjects consented via NCT00607230, NCT02081326,2012P002243, 2001P001379)

	**n**	**%Female**	**Age**	**AOO**	**Duration**
BCG – T1D	9	44	45 ± 3	26 ± 4	19 ± 3
Placebo – T1D	3	0	48 ± 3	29 ± 3	20 ± 3
Reference – T1D	40	47	40 ± 2	26 ± 2	13 ± 1
p-value (BCG-T1D vs Placebo – T1D)			*0.51*	*0.64*	*0.93*
p-value (BCG-T1D vs Reference – T1D)			*0.11*	*0.99*	*0.05*
p-value (Placebo-T1D vs Reference – T1D)			*0.07*	*0.53*	*0.15*

b. Clinical traits of the BCG treated and of the Placebo treated patients used to study the 8-year long term in vivo effects of the BCG vaccine (Fig 1, Fig 4c, Supplementary Table S2) (Subjects consented via NCT02081326, 2012P002243, 2001P001379)

	**n**	**%Female**	**Age**	**AOO**	**Duration**
BCG – T1D	3	33	36 ± 2	11 ± 6	25 ± 6
Placebo – T1D	3	0	48 ± 3	29 ± 3	20 ± 3
p-value			0.03	0.08	0.50

c. Clinical traits of the untreated type 1 diabetic and non-diabetic control subjects used in the Metabolomics Study for comparison to in vivo treated BCG or Placebo subjects (Fig. 3, Fig 5b, Supplementary Table S4) (Subject consented via 2001P001379)

	**n**	**%Female**	**Age**	**AOO**	**Duration**
Control - Nondiabetic untreated	50	56	39 ± 2		
T1D	106	40	34 ± 2	22 ± 2	8 ± 1
p-value			0.03		

d. Clinical traits of the type 1 diabetic subjects used in the RNAseq Study (Fig. 2b, Fig. 4a, Supplementary Table S1, Supplementary Fig S2) (Subject consented via 2001P001379)

	n	%Female	Age	AOO	Duration
T1D	3	33	30.3 ± 0.7	21.0 ± 4.0	9.3 ± 3.7

e. Clinical traits of the type 1 diabetic and non-diabetic subjects used in the lactate studies (Fig. 4d) (Subjects consented via NCT00607230, NCT02081326,2012P002243, 2001P001379)

	n	%Female	Age	AOO	Duration
Control-Nondiabetic untreated	10	70.0	37.6 ± 4.9		
T1D	23	52.2	34.0 ± 2.8	17.7 ± 2.7	16.3 ± 1.2
p-value			0.53		

f. Clinical traits of the type 1 diabetic and non-diabetic control subjects used in the additional studies (Fig. 4d, Supplementary Fig S2) (Subject consented via 2001P001379)

	n	%Female	Age	AOO	Duration
Control-Nondiabetic untreated	11	72.7	20.0 ± 4.5		
T1D	27	48.1	35.3 ± 2.7	20.0 ± 2.6	15.3 ± 1.3
p-value			0.83		

Supplementary Table S2

	Year 0	Year 5	Year 6	Year 7	Year 8
**HbA1c (%)**					
**T1D BCG Treated**	7.36±0.44	6.18±0.34	6.44±0.34	6.25±0.26	6.65±0.36
**T1D Placebo Treated**	7.10±0.55	7.07±0.41	7.18±0.62	7.17±0.46	7.22±0.38
**T1D Reference Population**	7.08±0.07	7.33±0.17			
**P-value**					
**BCG vs. Placebo**		**P=0.02**	**p=0.02**	**P=0.0008**	**P=0.0002**
**BCG vs. Reference**		**P=0.02**			
**Placebo vs Reference**		**P=0.73**			

For these 8 year followed clinical trial subjects: T1D BCG Treated (n=3), T1D placebo treated (n=3) and reference T1D population n=40)

Supplementary Table S3

X-axis#	FOXP3	TNFRSF18	IL2RA	IKZF2	IKZF4	CTLA4
1	cg01564333	cg00086243	cg09001761	cg00591406	cg00026033	cg05074138
2	cg01905377	cg02709725	cg09115275	cg01311718	cg00692047	cg05092371
3	cg02033323	cg04343794	cg11127249	cg01663232	cg01565774	cg08460026
4	cg04920616	cg06097659	cg11733245	cg01774108	cg03922997	cg14288266
5	cg06767008	cg07119157	cg13855852	cg02239891	cg17331199	cg22572158
6	cg08884343	cg07671976	cg16949914	cg05148385	cg20054248	cg24077172
7	cg10858077	cg08090128	cg26105232	cg07610406	cg20359445	cg26091609
8	cg15614573	cg08641866	cg26316423	cg08319019	cg20672549	
9	cg16350494	cg10583942	cg27131821	cg11217840	cg23363971	
10		cg12810734		cg11735605	cg24414325	
11		cg14886269		cg13779610	cg24828603	
12		cg15706223		cg14227558		
13		cg16046810		cg15488457		
14		cg18319381		cg16345559		
15		cg19237691		cg17384625		
16		cg19413397		cg20820997		
17		cg25725823		cg27225309		

Supplementary Table S4

Metabolites	Controls vs T1D		T1D vs T1D RX BCG		T1D vs T1D Placebo	
	p-value	*q-value*	*p-value*	*q-value*	*p-value*	*q-value*
**Purine Metabolites**						
Allantoin	0.027	0.208	0.107	0.002	0.188	0.240
Adenine	0.002	< 0.001	0.029	0.001	0.123	0.197
N1-Methyladenosine	< 0.001	< 0.001	0.083	0.002	0.036	0.097
N6-Carbamoylthreonyladenosine	0.003	<0.001	0.013	<0.001	0.084	0.159
7-Methylguanine	< 0.001	<0.001	0.014	<0.001	0.203	0.253
N2,N2-Dimethylguanosine	0.008	0.039	0.002	<0.001	0.012	0.050
**Pyrimidine Metabolites**						
Pseudouridine	< 0.001	0.001	0.057	0.002	0.292	0.302

Supplementary Figure S1

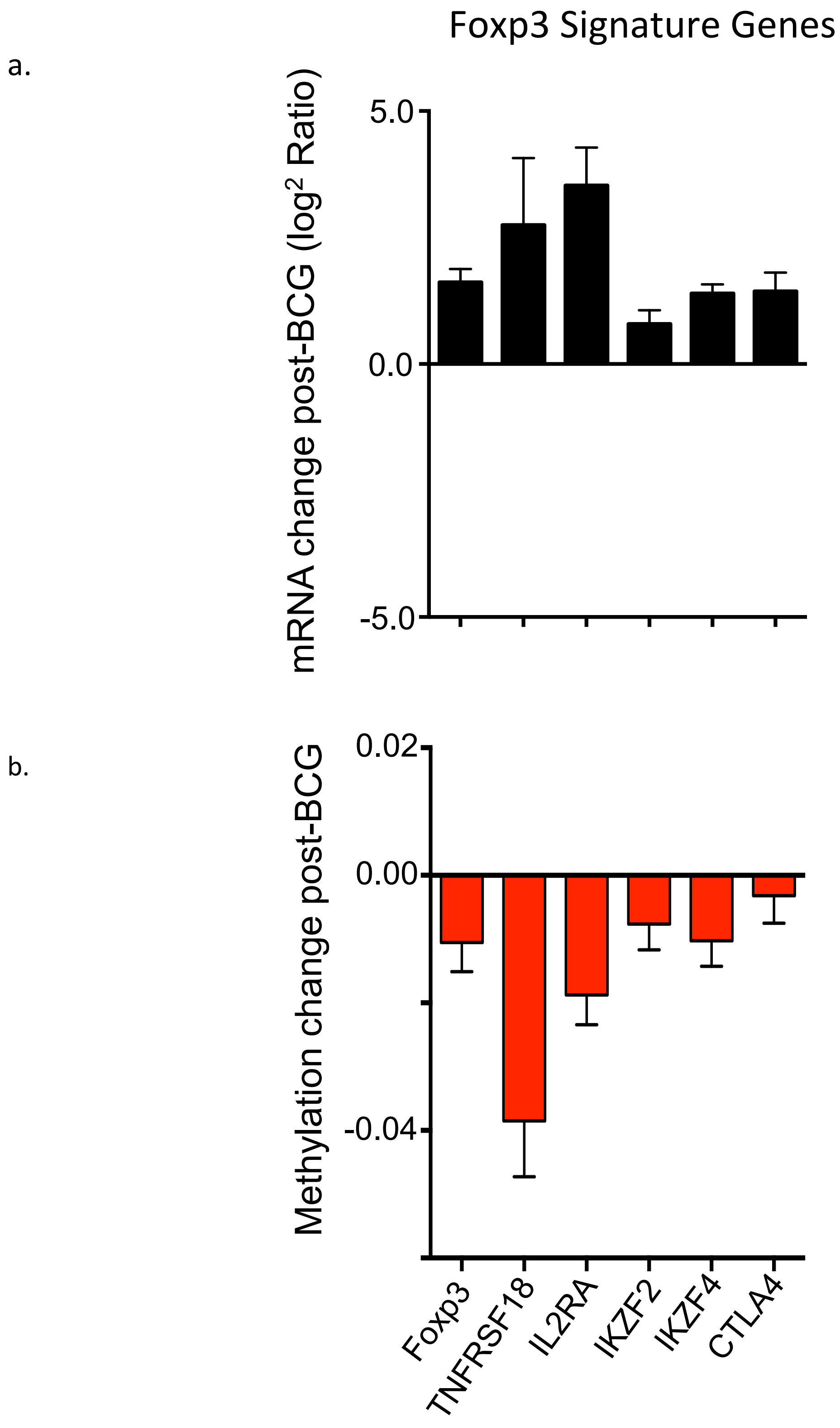

Supplementary Figure S2

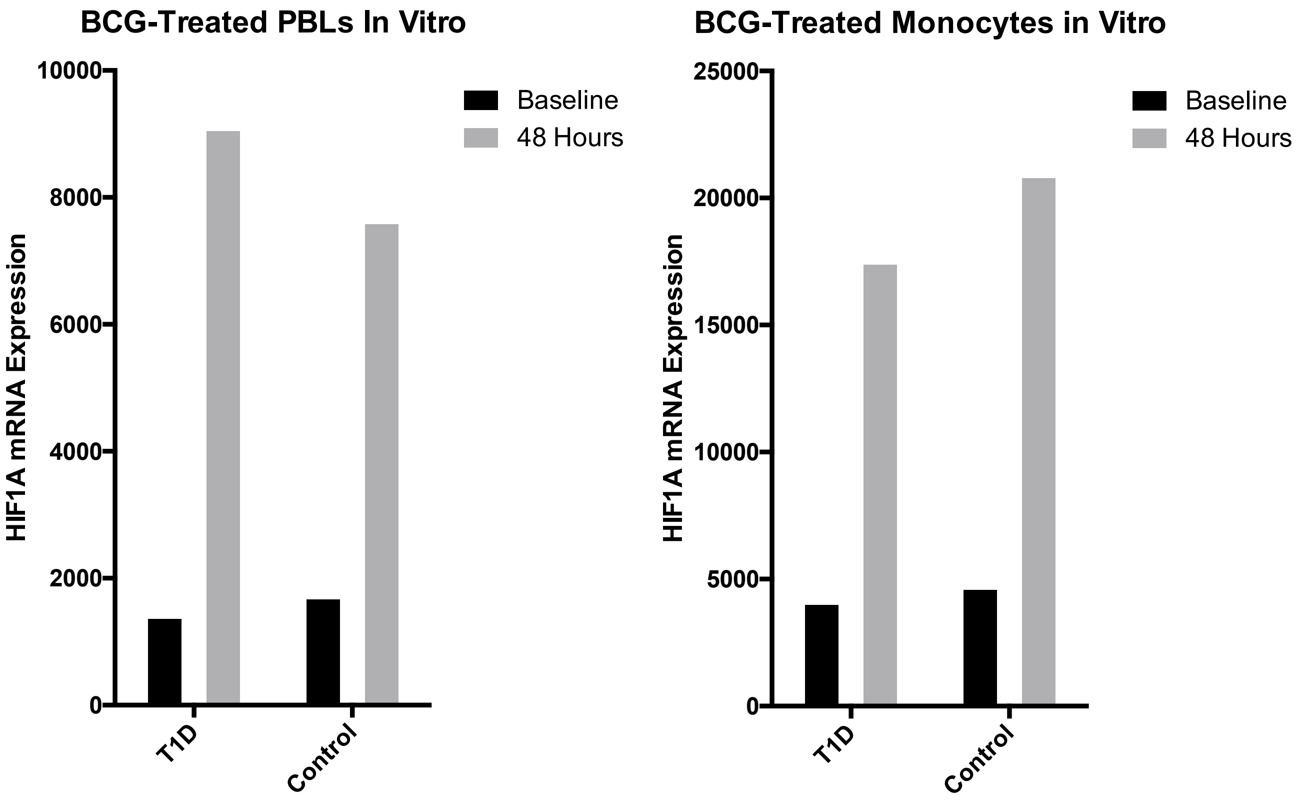

Supplementary Figure S3

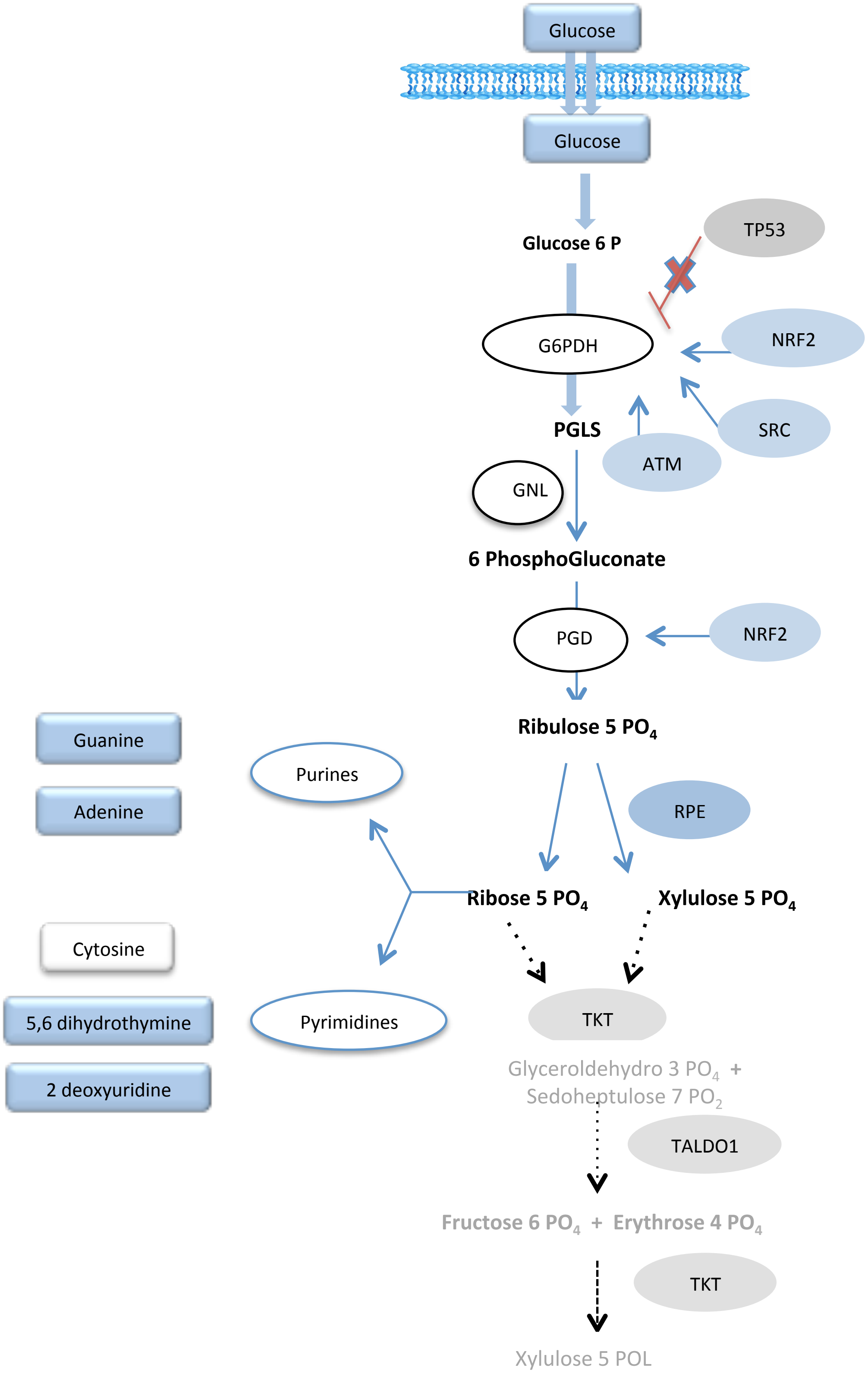

Supplement: Supplementary file 1 — Supplementary Materials [file 41541_2018_62_MOESM1_ESM.pdf]
